# Tadalafil increases the antitumor activity of 5-FU through inhibiting PRMT5-mediated glycolysis and cell proliferation in colorectal cancer

**DOI:** 10.1186/s40170-022-00299-4

**Published:** 2022-12-06

**Authors:** Yao Shen, Pan Zhao, Kewei Dong, Jiajia Wang, Huichen Li, Mengyang Li, Ruikai Li, Suning Chen, Yuxia Shen, Zhiyu Liu, Mianjiao Xie, Peng Shen, Jian Zhang

**Affiliations:** 1 The State Key Laboratory of Cancer Biology, Department of Biochemistry and Molecular Biology, The Fourth Military Medical University, Xi’an, 710032 China; 2 The Faculty of Hepatopancreatobiliary Surgery, The First Medical Center, Chinese People’s Liberation Army General Hospital, Beijing, China; 3 Department of Gastrointestinal Surgery, Xijing Hospital, The Fourth Military Medical University, Xi’an, 710032 China; 4 Department of Pharmacy, Xijing Hospital, The Fourth Military Medical University, Xi’an, 710032 China; 5 The State Key Laboratory of Cancer Biology, Institute of Digestive Diseases, Xijing Hospital, The Fourth Military Medical University, Xi’an, 710032 China; 6 Department of Experimental Surgery, Xijing Hospital, The Fourth Military Medical University, Xi’an, 710032 China; 7 Department of Oncology, Nanfang Hospital, Southern Medical University, Guangzhou, 510515 China

**Keywords:** Colorectal cancer, PRMT5, Tadalafil, 5-FU

## Abstract

**Background:**

Protein arginine methyltransferase 5 (PRMT5) is upregulated in multiple tumors and plays a pivotal role in cancer cell proliferation. However, the role of PRMT5 in colorectal cancer remains poorly understood.

**Methods:**

We detected the expression level of PRMT5 and glycolytic enzymes using online databases and colorectal cancer cell lines by immunohistochemical staining, quantitative real-time polymerase chain reaction (qRT-PCR), and western blotting. And MTT and colony formation assays were conducted to investigate cell proliferation. Then, we evaluated ECAR and OCR levels using a biological energy analyzer to investigate the energy status of colorectal cancer, and the transcriptional regulation was detected by dual luciferase reporter assay and ChIP assay. Finally, the efficacy of combined treatment of tadalafil and 5-FU was verified.

**Results:**

PRMT5 was highly expressed in colorectal cancer tissues compared with their normal counterparts and correlated with poor prognosis in CRC patients. Then, we demonstrated that PRMT5 knockdown or loss of function attenuated the viability of CRC cells, while overexpression of PRMT5 promoted cell proliferation. Mechanistically, PRMT5 enhanced glycolysis through transcriptionally activating LDHA expression. In addition, the PRMT5 inhibitor, tadalafil, rendered CRC cells sensitive to antitumor agent 5-FU in vitro and in vivo.

**Conclusions:**

Our data indicates that PRMT5 promoted colorectal cancer proliferation partially through activating glycolysis and may be a potential target for colorectal cancer therapy.

**Supplementary Information:**

The online version contains supplementary material available at 10.1186/s40170-022-00299-4.

## Background

Colorectal cancer (CRC) is one of the most common tumor types all over the world; despite the decline in CRC incidence, it still ranks the third in cancer-related death [1, 2]. A series of gene events caused colorectal carcinogenesis, which is involving complex mechanisms including cell proliferation, apoptosis, differentiation, and survival [3]. At early stage, surgery is the primary curative treatment for colorectal cancer, while chemotherapy is needed when the disease developed into an advanced stage or at a high probability of recurrence. 5-Fluorouracil (5-FU) played an important role in CRC chemotherapy in decades-years [4]. Although many advances exist in CRC chemotherapy, emerging resistance limited its application, tumor recurrence occurs, and the 5-year survival still remains a challenge [5–7]. Thus, combination with other targeted therapies might be necessary to improve the efficacy of 5-FU and benefit for CRC patients.

Recent studies showed that protein arginine methyltransferases (PRMTs) family played an important role in cancer progression [8]. As a type 2 PRMTs, PRMT5 has been found highly expressed and often correlated with poor prognosis in multiple cancers, including lung cancer, hepatocellular carcinoma, and breast cancer [9]. PRMT5 regulates the expression of target genes via symmetrically dimethylating of arginine on both histones and nonhistone proteins. For example, PRMT5 can catalyze the methylation of arginine 8 on histone H3 (H3R8) and arginine 3 on histone H4 (H4R3), resulting in the silencing of E2F1 [10], FBW7 [11], CCNE1 [12], and the metastasis inhibition factor Nm23 [13]. In addition, several studies indicated that PRMT5 promoted colorectal cancer progress [14–16]; however, the role of PRMT5 in colorectal cancer remains unclear [9], and the molecular mechanism of PRMT5 in the regulation of CRC cell growth and the related signaling axis is entirely unknown. Therefore, it is of vital importance to uncover the function of PRMT5 in colorectal cancer with an aim to identify novel prognostic and treatment targets.

In several decades, the most important theory in cancer metabolism area is the “Warburg effect” [17, 18], which provides cancer cells energy, building blocks, and suitable redox microenvironment when undergoing uncontrolled growth. Recent studies showed that PRMT5 has closely related to aerobic glycolysis through several mechanisms, such as repressing FBW7 and elevating c-Myc expression (11), regulating the LXRα/NF-κBp65 pathway [19]. However, there is not enough evidence to elucidate the major functions of PRMT5 in CRC cell metabolism, especially the relevant roles of PRMT5 in glycolysis pathway and whether PRMT5 sustains cancer tumorigenicity through regulating glycolytic enzymes have seldom been discussed.

In the present study, we demonstrated that the expression level of PRMT5 was elevated in CRCs, and PRMT5 promoted glycolysis-induced cell proliferation through transcriptionally activating LDHA. Finally, we found that the novel PRMT5 inhibitor tadalafil could enhanced the efficiency of 5-FU in CRC cells. Collectively, the results of our present study uncovered a novel PRMT5 role in colorectal cancer, and targeting PRMT5 might be a strategy for the improvement treatment of colorectal cancer.

## Methods

### Cell culture and reagents

The CRC cell lines used in this study include HCT116, HT29, SW480, and SW620. HCT116 and HT29 cells were cultured in McCoy’s 5A medium, and SW480 and SW620 were cultured in L15 medium supplemented with 10% fetal bovine serum (Invitrogen Gibco, USA). Cells were incubated at 37 °C, 5% CO_2_. All of the medium was added 10% fetal bovine serum. JNJ-64619178 (Selleck, cat. no. S8624), tadalafil (MCE, HY-90009A, Monmouth Junction, NJ, USA), 2-deoxy-D-glucose (2-DG) (MCE, cat. no. HY-13966), and oligomycin (MCE, cat. no. HY-N6782) were purchased from MedChemExpress.

### Clinical CRC samples and tissue microarray

Surgical resected cancerous colon tissues and their normal counterparts were collected by Xijing Hospital of The Fourth Military Medical University. They were stored in liquid nitrogen. In addition, all these patients did not receive any radio- or chemotherapy before surgery. Then, the total 103 paired tissue samples were positioned in a tissue microarray. Detailed information about this can be seen in a previous work [20]. All the patients have signed written informed consent, and this study was approved by the ethics committee of Xijing Hospital of The Fourth Military Medical University (reference no.: 2019-KY-30).

### Immunohistochemical staining (IHC)

Tissue microarrays were stained with primary antibodies against PRMT5 (cat. no. ab109451; Abcam). The Aperio ScanScope XT automated slide scanner was used to scan the tissue microarrays; after that, the expression levels of PRMT5 were scored semiquantitatively based on staining intensity and distribution using the immunoreactive score. Briefly, IHC score = SI (staining intensity) × PP (percentage of positive cells). SI was assigned as 0 = negative; 1 = weak; 2 = moderate; and 3 = strong. PP was defined as 0 = 0–15%; 1 = 15–50%; 2 = 50–75%; and 3 = 75–100%.

### Quantitative real-time polymerase chain reaction (qRT-PCR)

To detect mRNA expression, total RNA was extracted with TRIzol kit (Invitrogen, USA), and the complementary DNA was synthesized with the M-MLV First-Strand Kit (Life Technologies), followed by performing qPCR with the SYBR Green (Solarbio). Primer sequences used in the study were listed in Table S1. Relative expression of mRNA was evaluated by 2^−ΔΔCt^ method, and β-actin was used as an internal reference.

### Western blotting

Protein extracts were resolved through 10–15% SDS-PAGE, transferred to NC membranes, and probed with primary antibodies. Peroxidase-conjugated anti-mouse or rabbit antibody was used as secondary antibody, and the antigen–antibody reaction was visualized by enhanced chemiluminescence assay. The following commercial antibodies were used: β-actin (cat. no. 3700; Cell Signaling Technology), PRMT5 (cat. no. 79998; Cell Signaling Technology), LDHA (cat. no. 3582; Cell Signaling Technology), H3R8me2s (cat. no. ab272149; Abcam), and H4R3me2s (cat. no. ab194696; Abcam).

### 3-(4, 5-Dimethylthiazol-2-yl)-2, 5-diphenyltetrazoliumbromide (MTT) assay

Cells (1 × 10^3^ cells/mL) were seeded into 96-well plates and cultured for 24 h, 48 h, or 72 h. The MTT solution (100 μL, 0.5 mg/mL, Sigma) was added to each well, after incubating for 4 h. Then, discarding the culture medium, the purple crystals were dissolved with dimethyl sulfoxide (150 μL, Sigma). Next, the absorbance at 562 nm was evaluated by the microplate reader (Bio-Rad, Hercules, CA, USA).

### Colony formation assay

Cells were seeded into 6-well culture plates 500 or 1000 cells per well. After about 10 days of culture, we can see the obvious colony. Fixed the cells with 4% paraformaldehyde for 15 min, stained with 0.1% crystal violet for 20 min, and then washed the plate with water gently. Finally, use a microscope to count the number of colonies. Cell clusters which are more than 50 cells are counted as colony.

### In vitro* analysis of extracellular acidification rate (ECAR)*

The ECAR of cells were analyzed using the Seahorse XF Glycolysis Stress Test Kit (Seahorse Bioscience, Chicopee, MA, USA) on an XF24 Extracellular Flux analyzer (Seahorse Bioscience), and raw ECAR values were normalized to cell numbers. In short, the cells (5 × 10^5^) were seeded into Seahorse XF24 cell culture microplates. When pre-treated with the PRMT5 inhibitor 0.5 μM JNJ-64619178, 10 μM of tadalafil was added to the corresponding group 12 h before the assay started (or DMSO for control). For the ECAR analysis, glucose, oligomycin, and 2-DG were sequentially injected into each well at the specified time point. At last, the data were analyzed with the Seahorse XF-24 Wave software, and ECAR was presented in mpH/min. Experiments were performed in triplicate for three independent experiments.

### Glucose uptake assay

2-NBDG (Sigma) was used to determine glucose uptake according to the manufacturer’s protocols. In total, 5 × 10^5^ pretreated cells were plated into 6-well cell culture plates and incubated at 37 °C overnight. The next day, cells were starved for glucose for 2 h. After incubating with 50 μM 2-NBDG for 30 min, cells were then collected and used for determination of glucose uptake. The glucose uptake was measured by flow cytometry.

### Dual-luciferase reporter assay

To explore PRMT5 effect on the LDHA promoter transcriptional activity, the fragments of LDHA promoter were synthesized and inserted into the pGL3 vectors (Promega, Madison, WI, USA). The luciferase activity in the lysate of HCT116 cells co-transfected with a luciferase plasmid and pCDH-PRMT5 or pCDH-NC was evaluated by using a FLUOstar device (Omega Engineering, Deckenpfronn, Germany), with the Dual-Luciferase Reporter Assay System (Promega). Transfection efficiency was normalized by dividing the luciferase activity of the construct by the corresponding Renilla luciferase activity.

### Chromatin immunoprecipitation (ChIP) assay

A ChIP assay kit (Millipore, New Bedford, MA, USA) was used according to the manufacturer’s instructions. Cells cultured in a 10 cm dish were fixed with 4% formaldehyde to cross-link proteins to DNA. Chromatin was extracted from the cells using anti-H3R8me2s (cat. no. ab272149; Abcam), and cross-linked DNA was sheared into 250–500 bp fragments. IgG was used as negative control.

### In vivo *xenograft model*

To illustrate the effect of PRMT5 on tumor growth in vivo, 6-week-old male nude mice were used in our study. HCT116 cells were suspended in 100 μL PBS and injected into the right flank of mice subcutaneously to establish the colorectal cancer xenograft model. Nude mice were divided into four groups: control group, 5-FU group, tadalafil group, and combination group. 5-FU administration was at a concentration of 20 mg/kg body weight and intraperitoneal injection every other day, while tadalafil was given at 2 mg/kg body weight concentration by daily oral infusion. We monitored the tumor size every 3 days, and tumor volume (mm^3^) was estimated by the formula: tumor volume (mm^3^) = short diameter^2^ × long diameter/2. Three weeks after inoculation, tumor-bearing mice were sacrificed by cervical dislocation under anesthesia (2.5 mg/100 g body weight pentobarbital injection), and the subcutaneous xenograft tumors were excised. Finally, all tumors were kept in formalin for LDHA (cat. no.3582; Cell Signaling Technology) staining.

### Statistical analyses

Statistics were implemented with GraphPad Prism 5 software (GraphPad, San Diego, CA, USA). We obtained our data from at least three independent tests, and each test was conducted in triplicate. One representative experimental result was selected to display in present study from three or more independent experiments. Data were presented as the mean ± SEM. Unpaired Student’s *t*-test was applied to compare the difference between 2 groups. Correlation between PRMT5 and LDHA in CRC tissues was evaluated with the Pearson’s correlation analysis. Comparison of Kaplan–Meier survival curves was performed with the log-rank test. Difference was deemed significant if *P* < 0.05.

## Results

### PRMT5 was highly expressed in colorectal cancer and indicated a poor prognosis

**Fig. 1:**
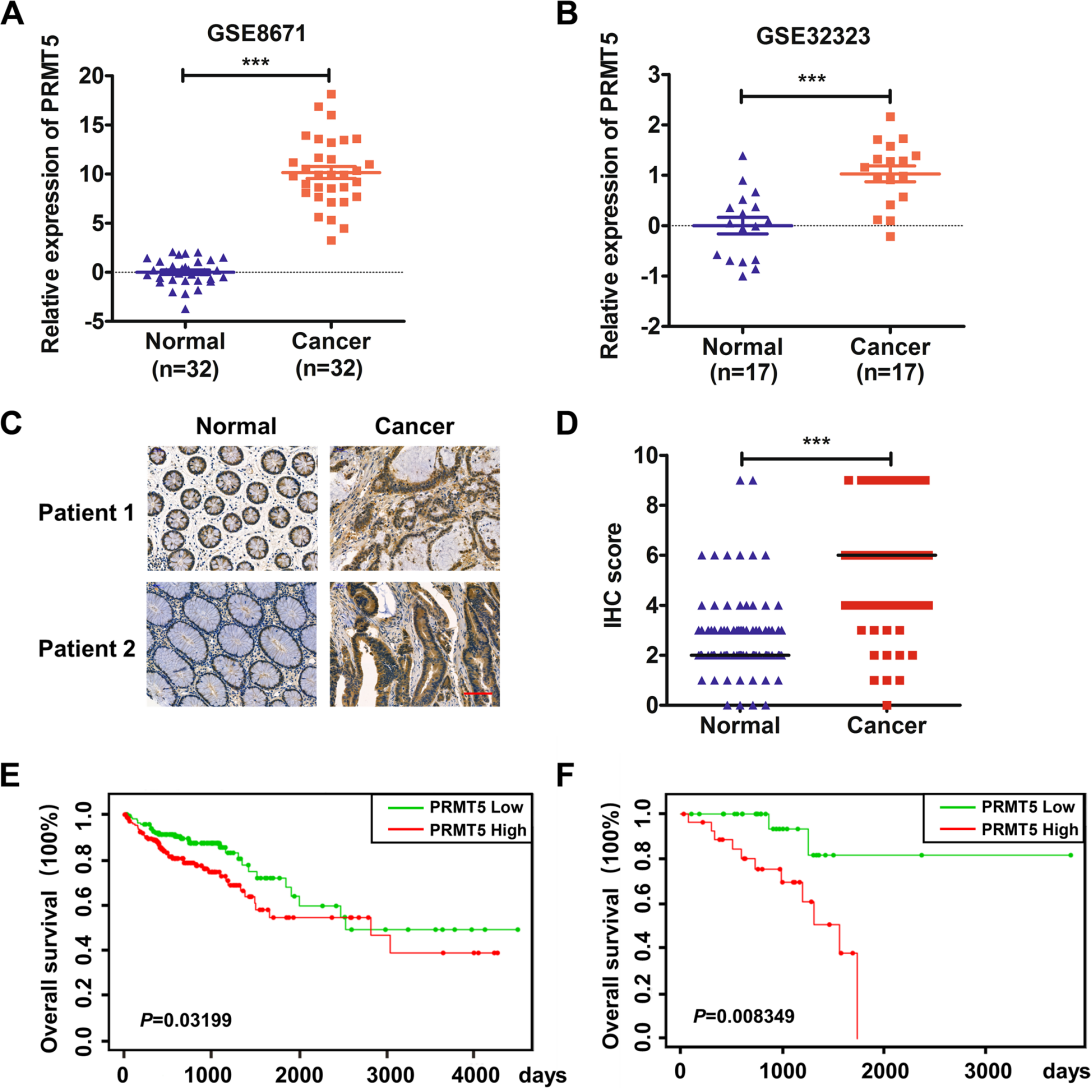
PRMT5 was highly expressed in colorectal cancer and indicated a poor prognosis. **A** and **B** Expression of *PRMT5* in CRC patients from GSE8671 (**A**) and GSE32323 (**B**). **C** Typical images of PRMT5 expression in CRC patients. **D** The expression levels of PRMT5 were scored semiquantitatively based on staining intensity and distribution in 103 paired CRC tissues positioned in a tissue microarray. Scale bar (red line) = 100 μm. **E** Survival analysis of PRMT5 in TCGA database (left, colon and rectum adenocarcinoma; right, rectum adenocarcinoma only). ***P < 0.001

We firstly analyzed the role of PRMT5 in CRC using TCGA database and two GEO datasets (GSE8671 and GSE32323). We found that PRMT5 expression was higher in colorectal tumors than the adjacent normal counterparts (Fig. 1 A and B and Fig. S1). Next, we measured the expression level of PRMT5 in tumor specimens from 103 patients by immunohistochemical staining, and our results demonstrated that PRMT5 localized both in nuclear and cytoplasm, and the expression level of PRMT5 was significantly higher in cancerous colon samples than that in adjacent normal tissues (Fig. 1 C and D). Finally, survival analysis suggested that higher PRMT5 expression correlated with worse prognosis in CRC patients (Fig. 1E). Collectively, these observations revealed that PRMT5 expression was upregulated in CRC and indicated the poor prognosis.

### PRMT5 promoted cell proliferation of colorectal cancer

**Fig. 2:**
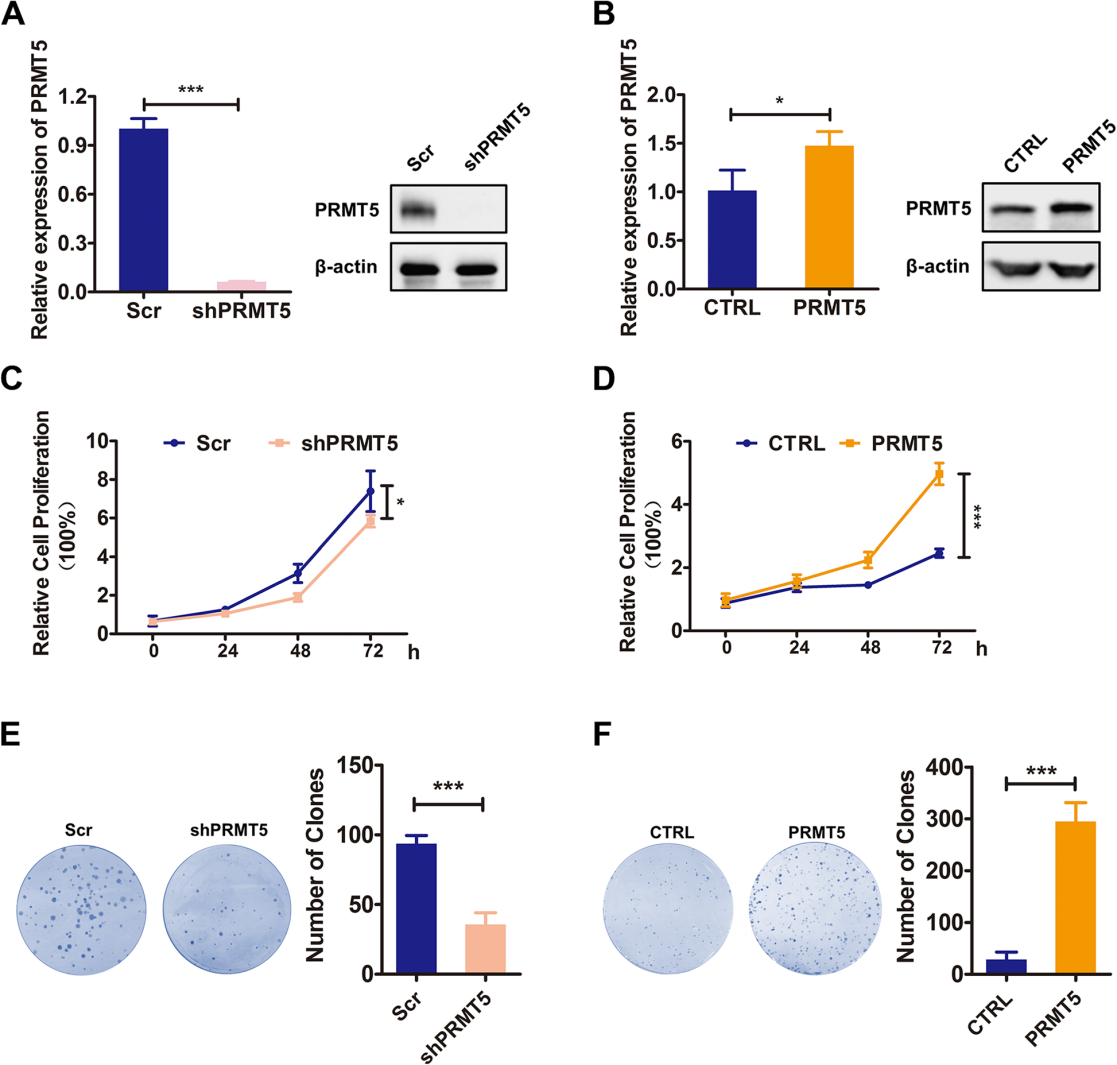
PRMT5 promoted cell proliferation of colorectal cancer. **A** and **B** qRT-PCR analysis of *PRMT5* mRNA expression levels and Western blotting analysis of PRMT5 protein levels in HCT116 scrambled (Scr) and PRMT5 knockdown (shPRMT5) (**A**) or EGFP (CTRL) and PRMT5-overexpressed cells (**B**). **C** and **D** % of cell proliferation of PRMT5-knockdown (**C**) and -overexpressed (**D**) HCT116 cells was evaluated by MTT assay. For PRMT5-KD group, 2000 cells were seeded per well, and PRMT5-OE group was seeded 1000 cells per well. Mean ± SEM of three independent experiments is shown. **E** and **F** Colony formation assays of PRMT5-knockdown (shPRMT5) (**E**) or PRMT5-overexpressed cells (**F**). Colony numbers were calculated, and statistical analysis was shown in the right panel of each image. *P < 0.05. ***P < 0.001

**Fig. 3:**
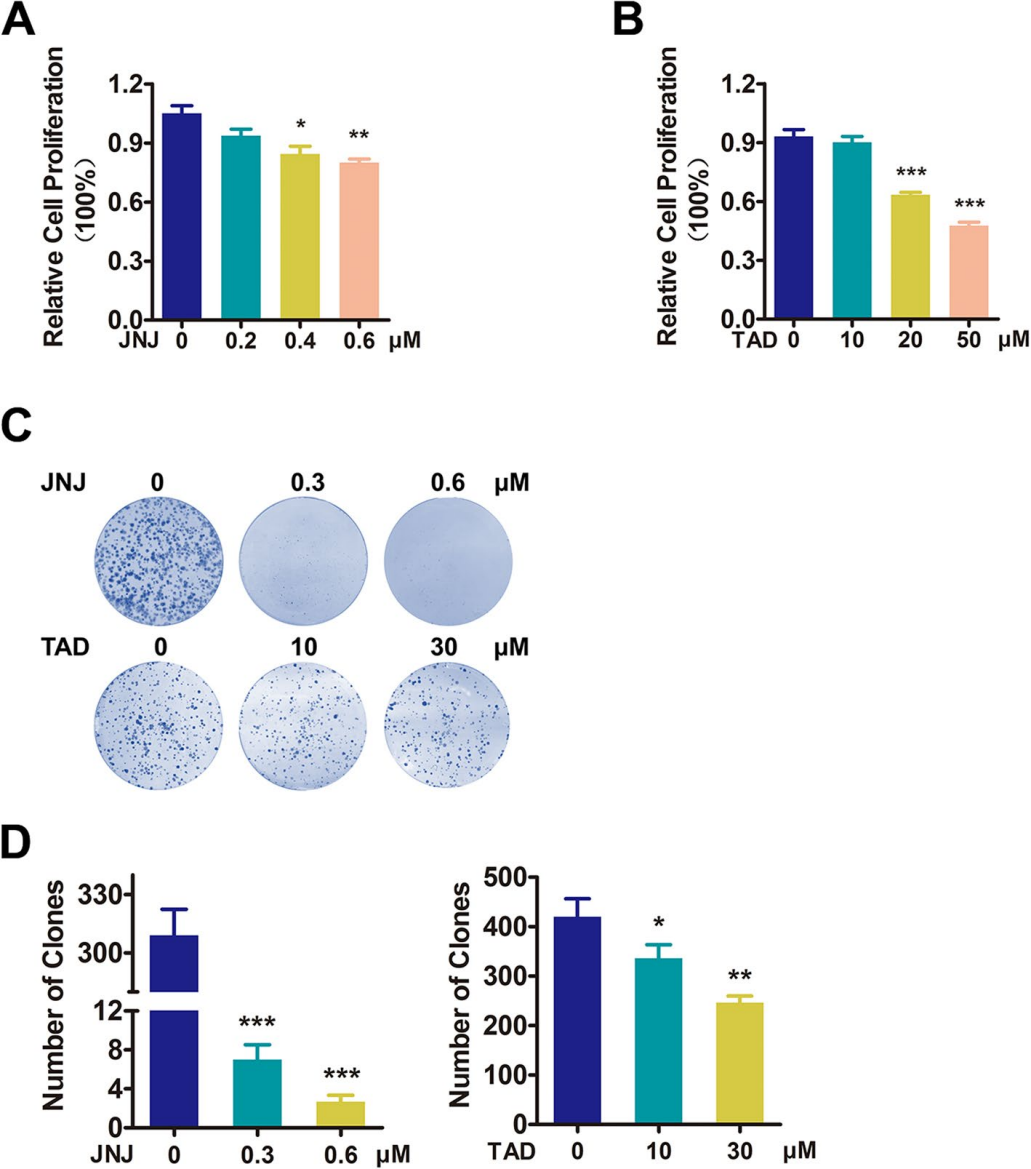
PRMT5 inhibitors suppress cell proliferation in CRC. **A** and **B** % of cell proliferation of HCT116 cells treated with **A** JNJ-64619178 (JNJ; 0.2, 0.4, 0.6 µM) or **B** tadalafil (TAD; 10, 20, 50 µM) for 24 h was evaluated by MTT assay. Mean ± SEM of three independent experiments is shown. **C** Colony formation assays of HCT116 cells pretreated with JNJ-64619178 (JNJ; 0, 0.3, 0.6 µM) or tadalafil (TAD; 0, 10, 30 µM). **D** Statistical analysis in (**C**). *P < 0.05; **P < 0.01; ***P < 0.001

To study the role of PRMT5 in colorectal tumorigenicity, firstly, we established the stable HCT116 cell lines with PRMT5 knockdown or overexpression, and we confirmed expression of PRMT5 by qRT-PCR and Western blotting analysis (Fig. 2 A and B), respectively. Then, we performed in vitro proliferation assays. As shown in Fig. 2 C and D, MTT assays demonstrated that silencing PRMT5 decreased the viability of HCT116 cells, while enhanced PRMT5 expression displayed a reverse phenotype. Similarly, the results of colony formation assays showed that silencing PRMT5 expression reduced the colony formation capacity, while overexpression of PRMT5 increased colony formation (Fig. 2 E and F). To further confirm the effect of PRMT5 on CRC cell proliferation, two PRMT5 inhibitors JNJ-64619178 and tadalafil [21] were used to treat CRC cells. Both inhibitors suppressed cell proliferation in a concentration-dependent manner (Fig. 3 A and B and Fig. S2 A and B) and could efficiently inhibit the function of PRMT5 (as indicated by H4R3me2s) (Fig. S2 C and D). Colony formation assays verified that PRMT5 blockade decreased cell proliferation of CRC (Fig. 3 C and D). Therefore, these data demonstrated that PRMT5 promoted colorectal cancer cell proliferation.

### Blockade of PRMT5 resulted in decreased glycolysis in colorectal cancer

**Fig. 4:**
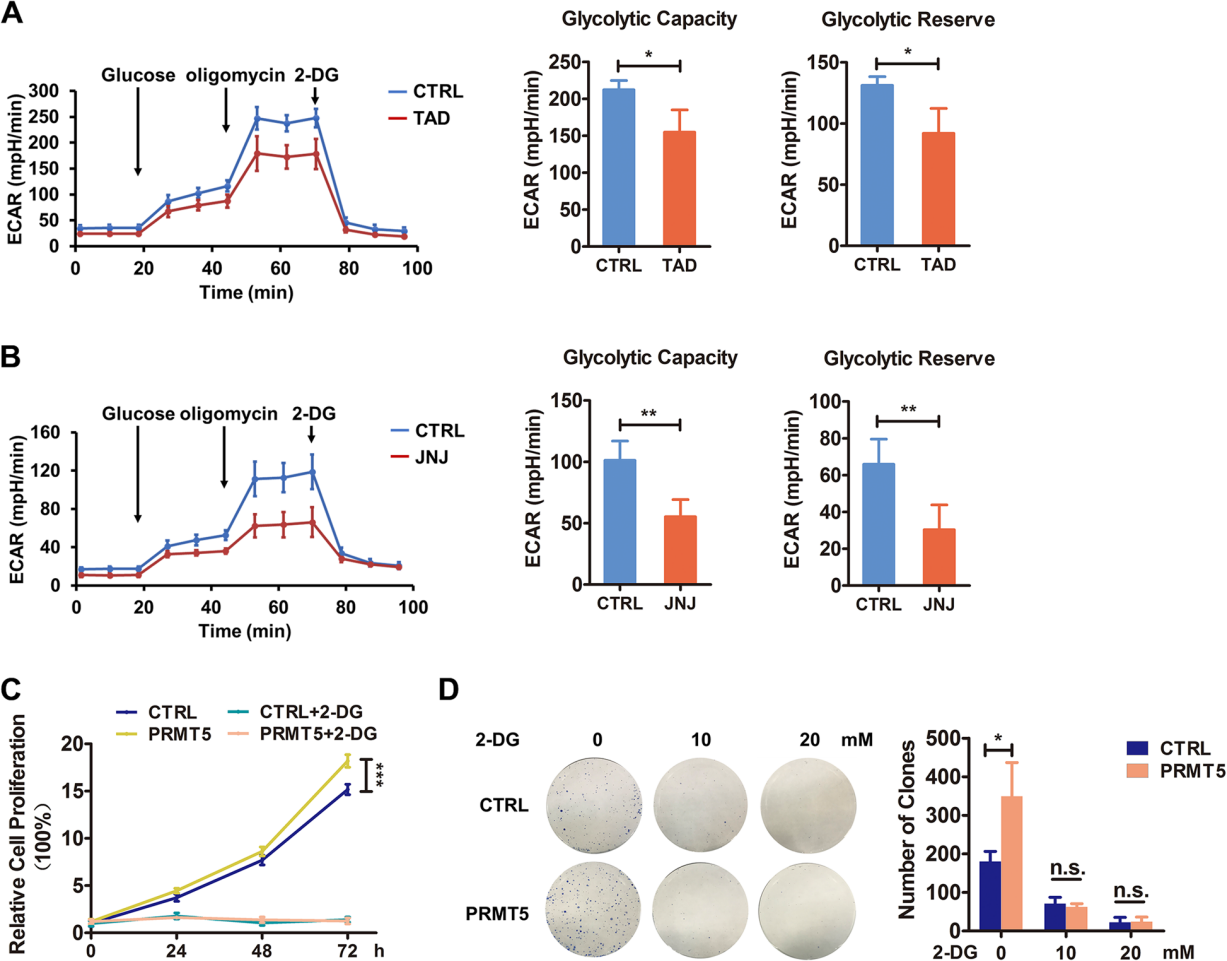
Blockade of PRMT5 resulted in decreased glycolysis in colorectal cancer. **A** and **B** ECAR analysis of HCT116 cells treated with tadalafil (TAD; 10 µM) (**A**) and SW620 cells treated with JNJ-64619178 (JNJ; 0.5 µM) (**B**). The glycolytic capacity and glycolytic reserve were shown. **C** 2-DG inhibited PRMT5-induced glycolysis. % of cell proliferation of PRMT5-overexpressed HCT116 cells treated with DMSO or 2-DG (10 mM) for 24 h was evaluated by MTT assay. Mean ± SEM of three independent experiments is shown. **D** Colony formation assays of EGFP (CTRL) and PRMT5-overexpressed HCT116 cells treated with 2-DG (0, 10, 20 mM), statistical analysis was showed in right panel. *P < 0.05; **P < 0.01; ***P < 0.001; n.s., no significance

Studies have elucidated that many cancer cells were dependent on aerobic glycolysis for the rapid progression. Thus, we performed ECAR measurements to explore whether PRMT5 affects glycolysis of CRC cells. Results showed that both JNJ-64619178 and tadalafil treatment inhibited glycolysis in colorectal cancer cells (Fig. 4 A and B and Fig. S3A). Moreover, compared with the corresponding control cells, PRMT5-knockdown HCT116 cells exhibited decreased glucose uptake (Fig. S3B). Next, to further confirm this finding, we used a glycolysis inhibitor 2-DG. As shown in Fig. 4 C and D, treating cells with 2-DG significantly abrogated the cell proliferation induced by PRMT5. In conclusion, PRMT5 could potentially regulate aerobic glycolysis in colorectal cancer.

### PRMT5 transcriptionally activated the expression of LDHA

**Fig. 5:**
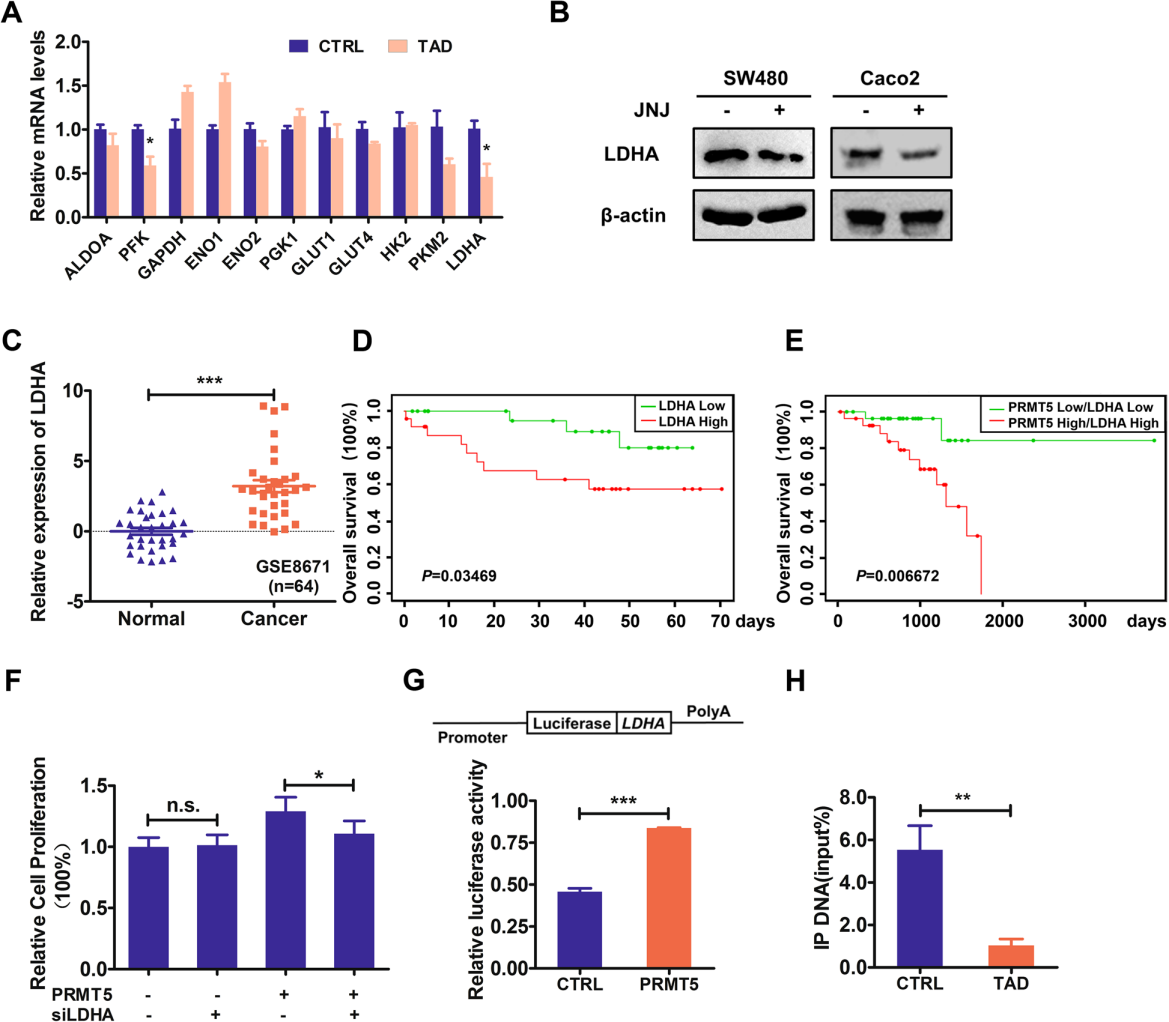
PRMT5 transcriptionally activated the expression of LDHA. **A** Fold change relative to control of glycolytic enzymes in HCT116 cells treated with or without tadalafil (TAD; 25 µM) for 24 h. Mean ± SEM of three independent experiments was shown. **B** Western blotting analysis to assess LDHA levels in HCT116, SW480, and Caco2 cells treated with or without JNJ-64619178 (JNJ; 0.5 μM). β-actin was used as loading control, and one representative experiment out of three is shown. **C** Expression of LDHA in CRC patients from GSE8671. **D** Survival analysis of LDHA in GSE17537 dataset. **E** Survival analysis of LDHA and PRMT5 in TCGA rectum adenocarcinoma. **F** % of cell proliferation of PRMT5-overexpressed HCT116 cells transfected with siNC or siLDHA was evaluated by MTT assay. Mean ± SEM of three independent experiments is shown. **G** The luciferase activity of LDHA promoter in EGFP (CTRL) and PRMT5-overexpressed HCT116 cells measured by dual luciferase reporter assay. **H** H3R8me2s binding to LDHA in PRMT5-overexpressed HCT116 cells treated with or without tadalafil (TAD; 25 µM) for 24 h measured by ChIP assay. *P < 0.05; **P < 0.01; ***P < 0.001; n.s., no significance

To address the mechanism of glycolysis reduction induced by PRMT5 blockade, qRT-PCR analysis was performed to search for targets of PRMT5. We found that the expressions of glycolytic enzymes were changed when PRMT5 activity was inhibited (Fig. 5A and Fig. S4 A–F). LDHA, a known key enzyme in glycolysis pathway, was obviously downregulated in PRMT5-inhibiting groups (Fig. 5B and Fig. S4 G and H). Additionally, in the GSE8673 cohort, we observed a higher level of LDHA expression in cancerous tissues than their adjunctive normal tissues (Fig. 5C). Moreover, bioinformation analysis demonstrated that higher LDHA (as well as LDHA and PRMT5 combination) indicated a worse prognosis in colorectal cancer patients (Fig. 5 D and E).

To further explore the mechanism in PRMT5-induced cell proliferation, we knocked down LDHA using siRNAs (Fig. S4I), although we did not detect obvious alteration of cell proliferation by LDHA silencing per se (Fig. S4J), the cell proliferation showed a significant drop in PRMT5 overexpressing group (Fig. 5F). Then, we measured the impact of PRMT5 on LDHA promoter activity; the dual-luciferase assay results demonstrated that PRMT5 could suppress LDHA promoter activity (Fig. 5G). By performing ChIP assay, we found that overexpressed PRMT5 in HCT116 cells increased the occupancy of heterochromatin markers H3R8Me2s on the LDHA promoter (Fig. 5H). Collectively, these results suggest that PRMT5 could epigenetically activate LDHA expression in CRC cancer.

### Tadalafil enhanced the efficacy of 5-FU in CRC cells

**Fig. 6:**
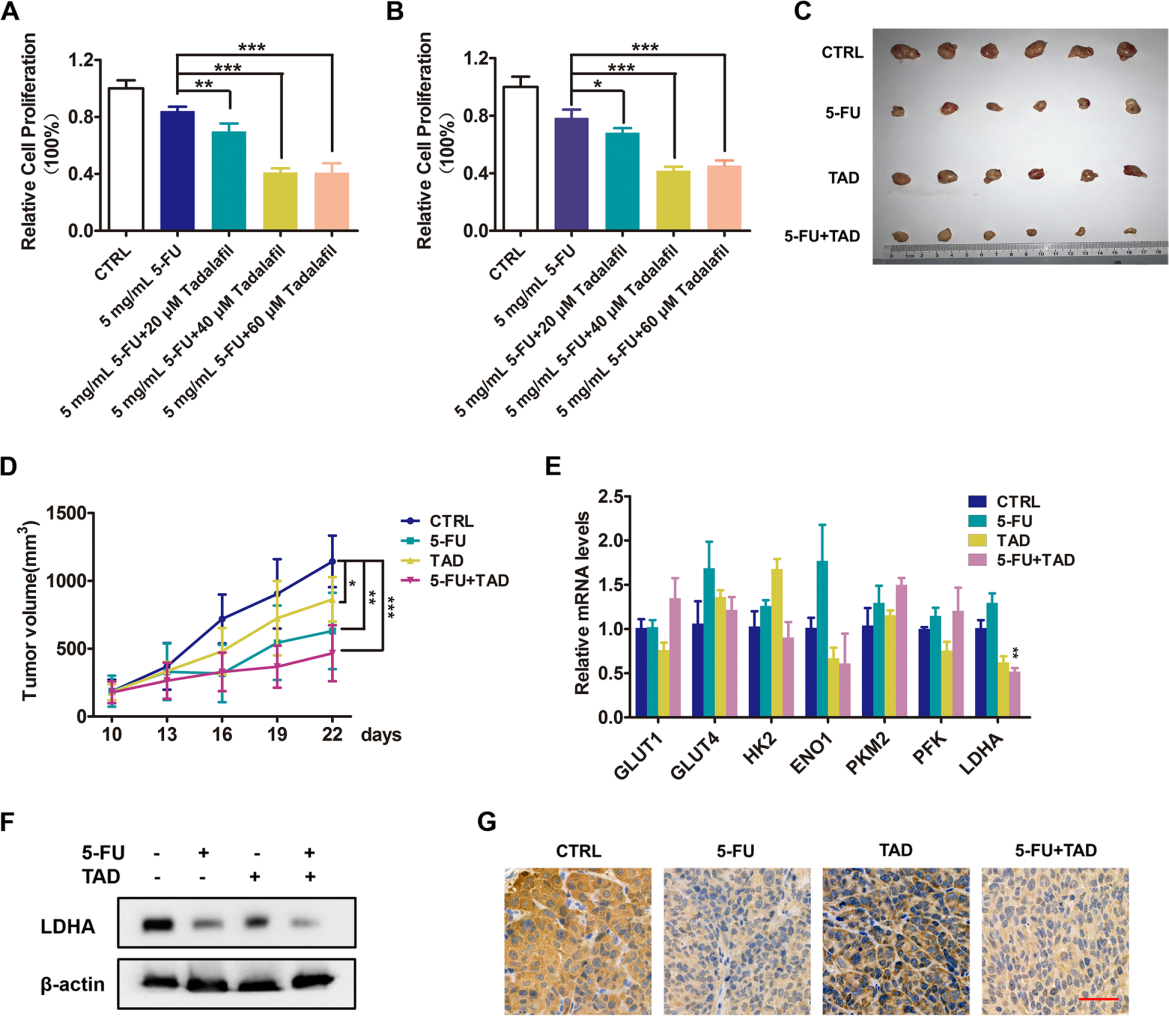
Tadalafil enhanced the efficacy of 5-FU in CRC cells. **A** and **B** % of cell proliferation of SW480 (**A**) and HCT116 (**B**) treated with 5 mg/mL 5-FU and 0, 20, 40, and 60 μM tadalafil for 12 h was evaluated by MTT assay. Mean ± SEM of three independent experiments is shown. **C**–**G** Colorectal cancer xenograft model in nude mice. Nude mice were divided into four groups: control group, 5-FU group, tadalafil group, and combination group. *n* = 6. **C** Tumor pictures of nude mice. **D** Tumor volume growth curve; tumor volume (mm.^3^) = short diameter2 × long diameter/2. **E** Fold change relative to control of glycolytic enzymes in tumor samples. Mean ± SEM of three independent experiments is shown. **F** Western blotting analysis to assess LDHA levels in tumor samples. β-actin was used as loading control, and one representative experiment out of three is shown. **G** IHC staining of LDHA in tumor samples. Scale bar (red line) = 50 μm. *P < 0.05; **P < 0.01; ***P < 0.001

Our previous work found that the FDA-approved drug tadalafil could act as a novel PRMT5 inhibitor and suppress tumor growth in breast cancer; we aimed to verify whether tadalafil could be used in colorectal cancer treatment. Cell proliferation assays in vitro implied that combination of tadalafil with 5-FU could inhibit cell growth of CRC (Fig. 6 A and B). Finally, to verify the results, we established a xenograft model in nude mice. Observations revealed that the tumor volume and mass of nude mice were remarkably reduced after administrated with tadalafil and 5-FU combination, indicating that blockade of PRMT5 activity could inhibit the cell growth and increase the efficacy of 5-FU in colorectal cancer cells (Fig. 6 C and D). In addition, qRT-PCR and IHC staining showed that the expression of LDHA was significantly declined in combination group, establishing that PRMT5 blockade can inhibit the growth of colorectal cancer via the LDHA regulation (Fig. 6 E–G).

## Discussion

Colorectal cancer ranks the third in the malignant cancers worldwide [1]. Adjuvant chemotherapy played a pivotal role in CRC therapy, but their efficacy is limited to a portion of patients. Therefore, new targets are needed in order to improve the prognosis of CRC patients. Here, PRMT5 was found to be upregulated in colorectal cancer and could promote cell proliferation by transcriptionally activating LDHA to enhance aerobic glycolysis, suggesting that PRMT5 may be a potential therapeutic target for colorectal cancer.

PRMT5 is a type 2 PRMTs, which catalyzes the symmetric dimethylation of arginine residues on histone and nonhistone proteins. At present, there are relatively few studies on the relation of PRMT5 with colorectal cancer. In the present study, TCGA data analysis showed an elevated PRMT5 expression in colorectal cancer tissues; this result was further validated in another two GSE datasets and clinical CRC tumor tissues. In addition, high PRMT5 expression implies a poor prognosis in CRC patients. These findings suggest that PRMT5 played an important role in CRC and may serve as a prognostic marker. Then, we knocked down and overexpressed PRMT5 in colorectal cancer cells respectively. Data indicated that PRMT5 promotes cell proliferation in CRC, which is consistent with other studies [9].

Metabolic reprograming represents a hallmark of cancer cells. In the last decade, a large number of studies has elucidated that oncogenes and tumor suppressors rewire metabolic pathways to provide cancer cells energy, biosynthetic precursors, and reducing equivalents they need. In recent years, more and more studies have revealed that cancer cells alter their metabolic phenotype to a glycolytic mode to meet their demands in malignant progression. Our previous data showed that metabolic profiling in colorectal cancer tissue is very different from their normal counterparts, the alteration manifests in energy metabolism, amino acid metabolism, glutathione metabolism, etc. [22]. In current study, we found that PRMT5 blockade attenuated glycolysis (as indicated by ECAR level) in CRC cells through transcriptionally activating LDHA, indicating that PRMT5 blockade may prevent the growth of CRC cells by inhibiting glycolysis, although many works have elucidated that glycolysis promotes cell growth and invasion in cancers. However, as a key enzyme in glycolysis, no alteration was observed in CRC cell proliferation when we knocked down LDHA. Unlike another study, LDHA inhibitor oxamate inhibited protumorigenic cascades in other cancer types [23]. It prompts us to carry out more experiments to further analyze the detailed mechanism.

In the final stage of this study, we conducted both in vitro and in vivo experiments to explore the possibility of PRMT5 to be a therapeutic target. Subcutaneous tumor model in nude mice showed that PRMT5 inhibitor tadalafil could enhance the antitumor efficacy of 5-FU. These experiments suggested that PRMT5 may be a therapeutic target in CRC treatment, as tadalafil is already been approved by FDA for erectile dysfunction and pulmonary arterial hypertension (PAH) treatment [24]. Our data provided the feasible approach for tadalafil used for colorectal cancer treatment in clinic.

This study demonstrated the function of PRMT5 in CRC, but there are some limitations. First, we need more clinical samples to verify the expression and prognostic function of PRMT5 in CRC. Second, tadalafil enhanced the effectiveness of 5-FU in CRC cells, but we did not explore how is the function mode. Thus, further studies relating to this are needed.

## Conclusions

Our work indicates that PRMT5 promotes cell proliferation of CRC by regulating glycolysis. What’s more, blockade of PRMT5 with tadalafil inhibits the growth of CRC cells and enhances 5-FU’s antitumor effect. Therefore, PRMT5 may be a potential target for CRC treatment.

## Supplementary Information

**Additional file 1.**

**Additional file 2.**

**Additional file 3.**

## Data Availability

All data generated or analyzed during this study are included in this published article and its supplementary information files (see Additional file 1 — supplementary).

## Abbreviations

ChIP: Chromatin immunoprecipitation
2-DG: 2-Deoxy-D-glucose
5-FU: 5-Fluorouracil
CRC: Colorectal cancer cells
ECAR: Extracellular acidification rate
IHC: Immunohistochemical staining
LDHA: Lactate dehydrogenase A
MTT: 3-(4,5-Dimethylthiazol-2-yl)-2,5-diphenyltetrazolium bromide
OCR: Oxygen consumption rate
PRMT5: Protein arginine methyltransferase 5
qRT-PCR: Quantitative real-time polymerase chain reaction
