# Supplementary material for: Tadalafil increases the antitumor activity of 5-FU through inhibiting PRMT5-mediated glycolysis and cell proliferation in colorectal cancer
Source: Cancer Metab. 2022 Dec 6;10:22. doi: 10.1186/s40170-022-00299-4 (PMC9727889; doi:10.1186/s40170-022-00299-4)
Supplement: Supplementary file 1 — Additional file 1. [file 40170_2022_299_MOESM1_ESM.docx]

**Table S1.** Primer sequences used in this study.

| **Primer** | **Forward（5’-3’）** | **Reverse（5’-3’）** |
| --- | --- | --- |
| β-ACTIN | CATGTACGTTGCTATCCAGGC | CTCCTTAATGTCACGCACGAT |
| PRMT5 | CTGTCTTCCATCCGCGTTTCA | GCAGTAGGTCTGATCGTGTCTG |
| ALDOA | AGGCCATGCTTGCACTCAGAAGT | AGGGCCCAGGGCTTCAGCAGG |
| GLUT1 | CATCCCATGGTTCATCGTGGCTGAACT | GAAGTAGGTGAAGATGAAGAACAGAAC |
| HK2 | GAGCCACCACTCACCCTACT | CCAGGCATTCGGCAATGTG |
| LDHA | ATGGCAACTCTAAAGGATCAGC | CCAACCCCAACAACTGTAATCT |
| PFK | GGAGAAGCTGCGCGAGGTTTAC | ATTGTGCCAGCATCTTCAGCATGAG |
| PKM2 | GCCCGTGAGGCAGAGGCTGC | TGGTGAGGACGATTATGGCCC |
| PGK1 | ATGTCGCTTTCTAACAAGCTGA | GCGGAGGTTCTCCAGCA |
| ENO1 | GACTTGGCTGGGCAACTCTG | GGTCATCGGGAGACTTGAA |
| ENO2 | TCATGGTGAGTCATCGCTCAGGAG | ATGTCCGGCAAAGCGAGCTTCATC |
| GLUT4 | TTTTGAGATTGGCCCTGGCCCCAT | CTCAGGTACTCTTAAGAAGGTGAAG |
| GAPDH | TTCCGTGTCCCCACTGCCAACGT | CAAAGGTGGAGGAGTGGGTGTCGC |


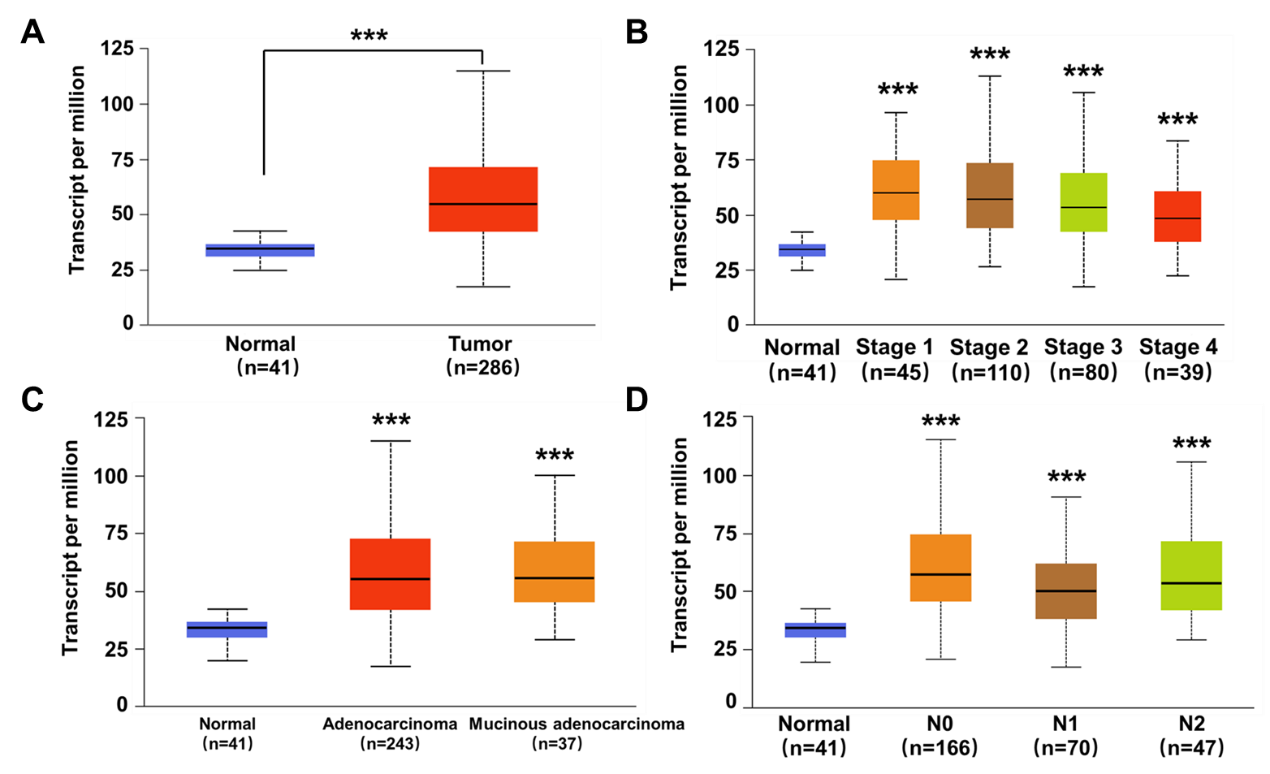


**Figure S1.** Expression of *PRMT5* in CRC patients from TCGA database. **(A)** PRMT5 expression was increased in colorectal adenocarcinoma compared with normal tissues; **(B)**  PRMT5 expression was significantly up-regulated in colorectal adenocarcinoma at all stages compared with normal tissue; **(C)** PRMT5 expression was up-regulated in adenocarcinoma and mucous adenocarcinoma tissues; **(D)** PRMT5 expression was up-regulated in all metastatic stages of colorectal adenocarcinoma compared with normal tissues.


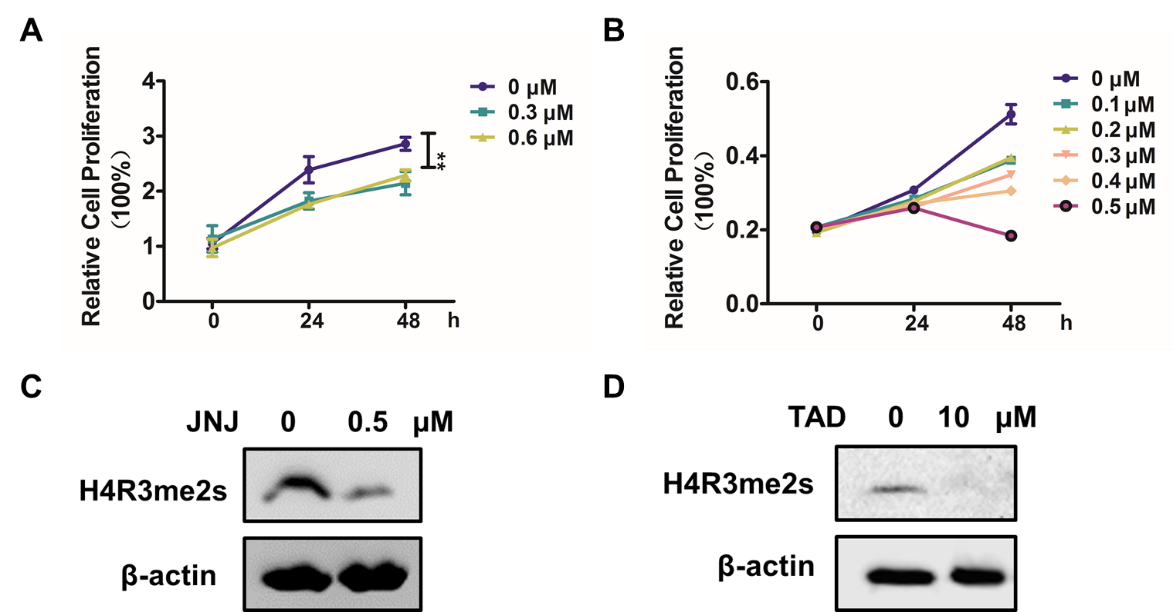


**Figure S2.** PRMT5 inhibitors suppress cell proliferation in CRC. **(A)** % of cell viability of HCT116 cells treated with JNJ-64619178 (0, 0.3, 0.6 µM) for 0, 24, 48 h was evaluated by MTT assay. Mean ± SEM of three independent experiments is shown. **(B)** % of cell viability of SW620 cells treated with JNJ-64619178 (0, 0.1, 0.2, 0.3, 0.4, 0.5 µM) for 0, 24, 48 h was evaluated by MTT assay. Mean ± SEM of three independent experiments is shown. **(C, D)** Western blotting analysis to assess H4R3me2s levels in HCT116 cells treated with or without **(C)** JNJ-64619178 (JNJ; 0.5 μM) and **(D)** tadalafil (TAD; 10 µM). β-actin was used as loading control and one representative experiment out of three is shown. (**) *P* < 0.01.


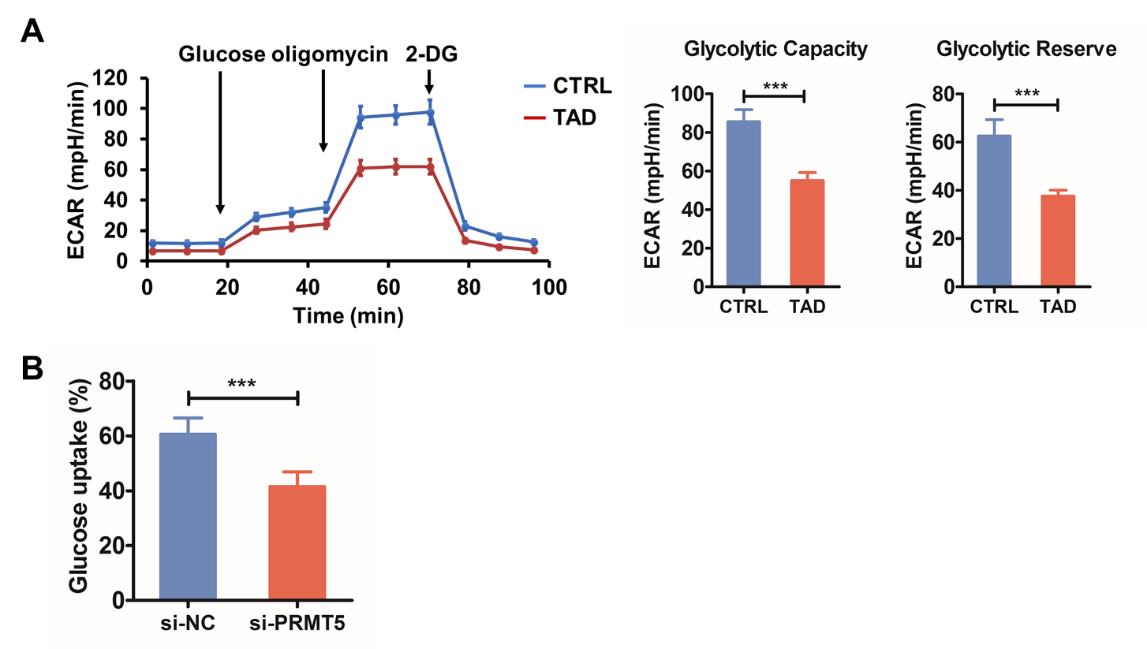


**Figure S3.** Blockade of PRMT5 resulted in decreased glycolysis in colorectal cancer. (A) ECAR analysis of SW480 cells treated with tadalafil (TAD; 10 µM). The glycolytic capacity and glycolytic reserve were shown. **(B)** glucose uptake of HCT116 cells transfected with siNC or siPRMT5. Mean ± SEM of three independent experiments is shown. (***) *P* < 0.001.


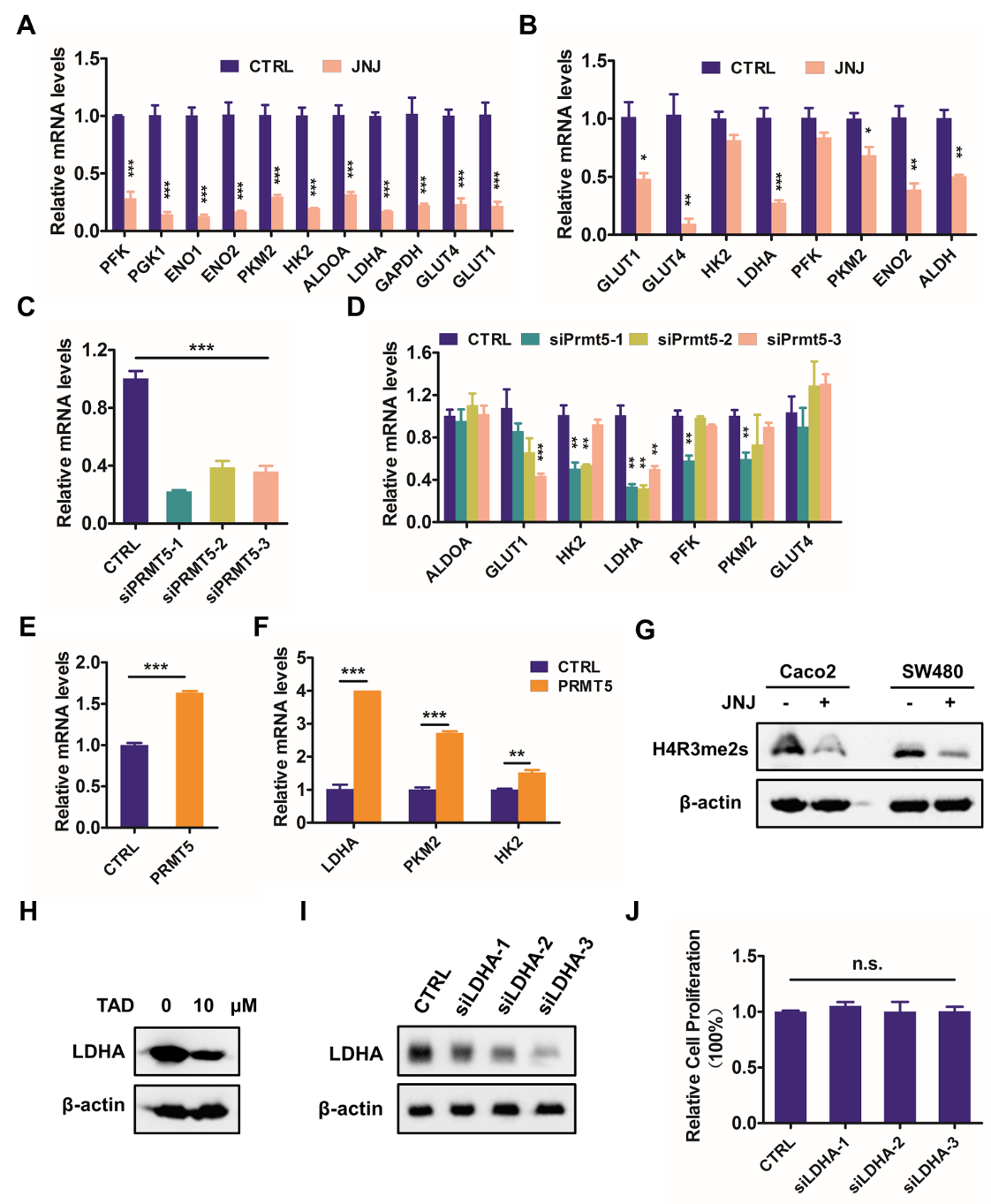


**Figure S4.** PRMT5 transcriptionally activated the expression of LDHA. **(A, B)** Fold change relative to control of glycolytic enzymes in **(A)** HCT116 cells and **(B)** SW480 cells treated with or without JNJ-64619178 (JNJ; 0.5 µM) for 24 h. Mean ± SEM of three independent experiments was shown. **(C)** qRT-PCR analysis of *PRMT5* mRNA expression of HCT116 cells transfected with siNC (CTRL), siPRMT5-1，siPRMT5-2 and siPRMT5-3 **. (D)** Fold change relative to control of glycolytic enzymes in HCT116 cells transfected with siNC (CTRL), siPRMT5-1，siPRMT5-2 and siPRMT5-3**. (E)** qRT-PCR analysis of *PRMT5* mRNA expression in SW480-EGFP (CTRL) and PRMT5-overexpressed cells. **(F)** Fold change relative to control of glycolytic enzymes in SW480-EGFP (CTRL) and PRMT5-overexpressed cells. **(G)** Western blotting analysis to assess H4R3me2s levels in SW480 and Caco2 cells treated with or without JNJ-64619178 (JNJ; 0.5 μM). β-actin was used as loading control and one representative experiment out of three is shown. **(H)** Western blotting analysis to assess LDHA levels in HCT116 cells treated with or without tadalafil (TAD; 10 µM). β-actin was used as loading control and one representative experiment out of three is shown. **(I)** Western blotting analysis to assess LDHA levels in HCT116 cells transfected with siNC (CTRL), siLDHA-1，siLDHA-2 and siLDHA-3**.** β-actin was used as loading control and one representative experiment out of three is shown. **(J)** % of cell viability of HCT116 cells transfected with siNC (CTRL), siLDHA-1，siLDHA-2 and siLDHA-3 was evaluated by MTT assay. Mean ± SEM of three independent experiments is shown. (*) *P* < 0.05; (**) *P* < 0.01; (***) *P* < 0.001; (n.s.) no significance.
