# Supplementary material for: Tadalafil increases the antitumor activity of 5-FU through inhibiting PRMT5-mediated glycolysis and cell proliferation in colorectal cancer
Source: Cancer Metab. 2022 Dec 6;10:22. doi: 10.1186/s40170-022-00299-4 (PMC9727889; doi:10.1186/s40170-022-00299-4)
Supplement: Supplementary file 2 — Additional file 2. [file 40170_2022_299_MOESM2_ESM.docx]

**Original blotting image**


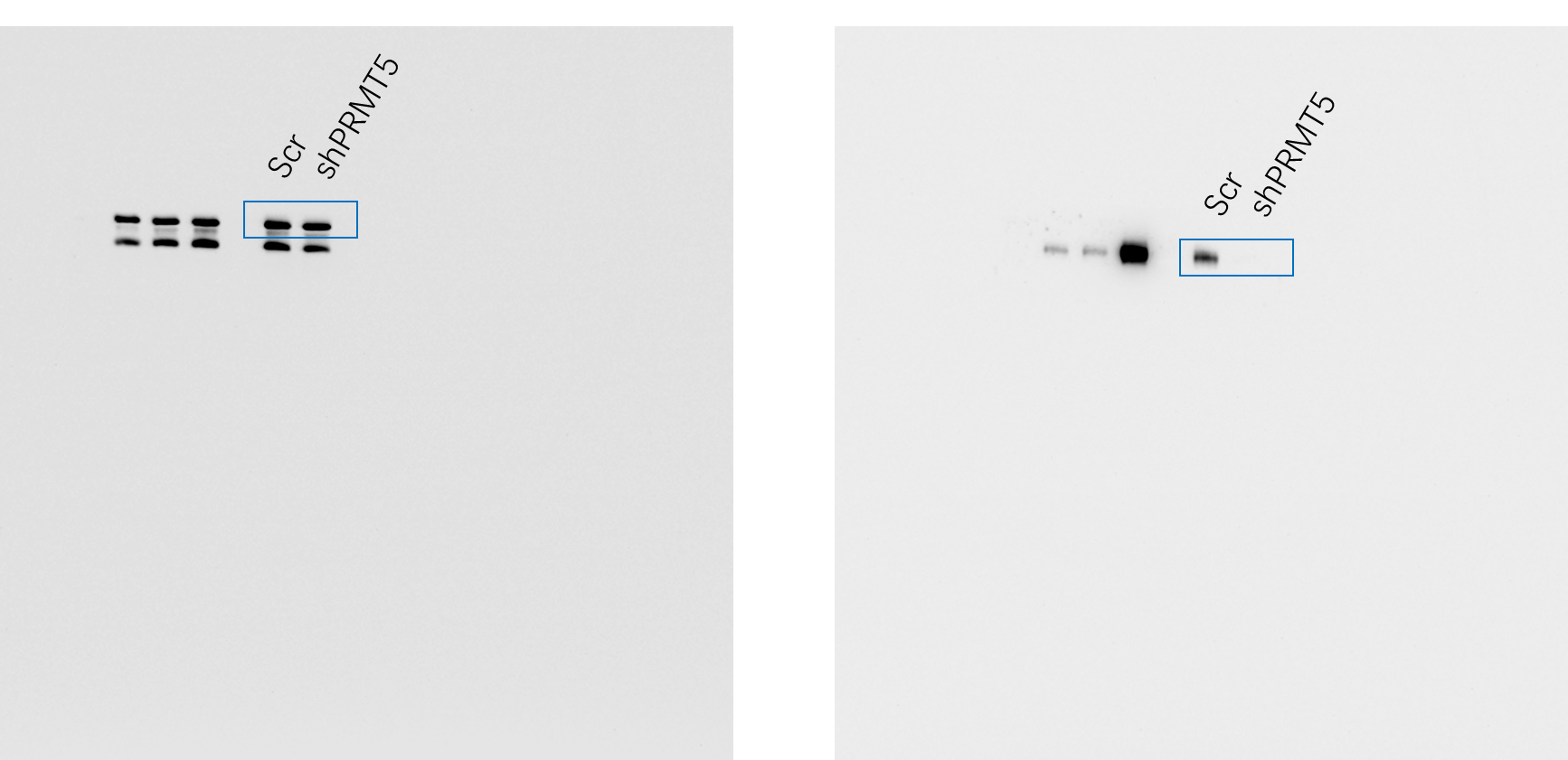


Figure 2A Original blotting of PRMT5 knockdown in HCT116 cells.

Left: anti-β-actin; Right: anti-PRMT5


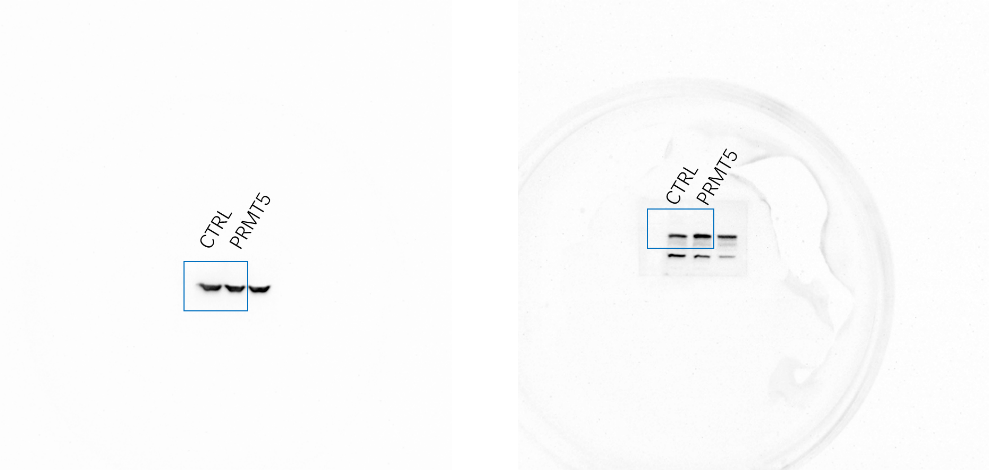


Figure 2B Original blotting of PRMT5 overexpression in HCT116 cells.

Left: anti-β-actin; Right: anti-PRMT5


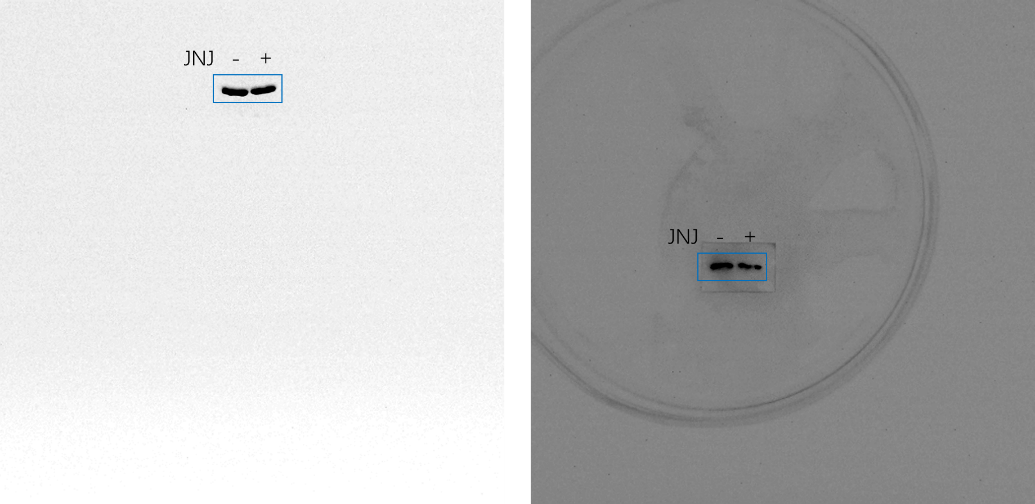


Figure 5B Original blotting of LDHA expression in sw480 cells.

Left: anti-β-actin; Right: anti-LDHA

**
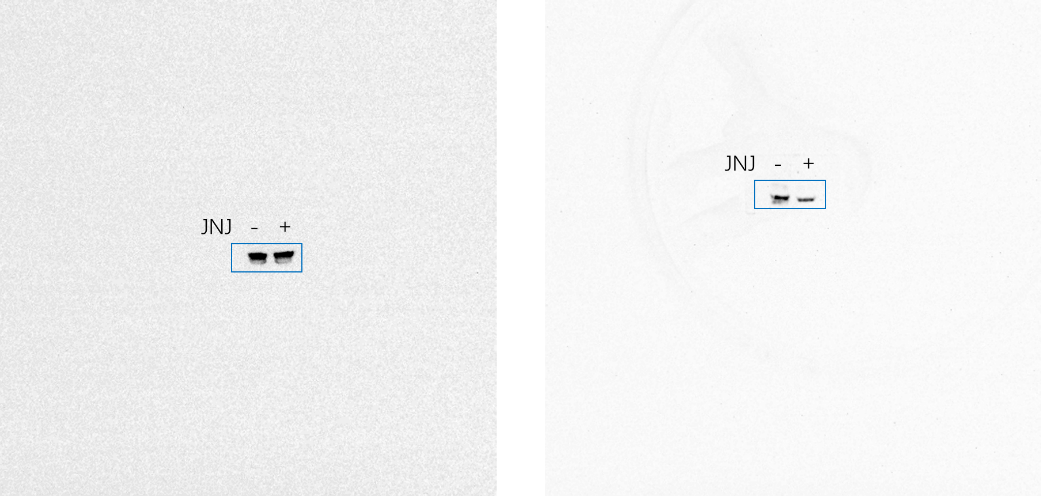
**

Figure 5B Original blotting of LDHA expression in Caco2 cells.

Left: anti-β-actin; Right: anti-LDHA


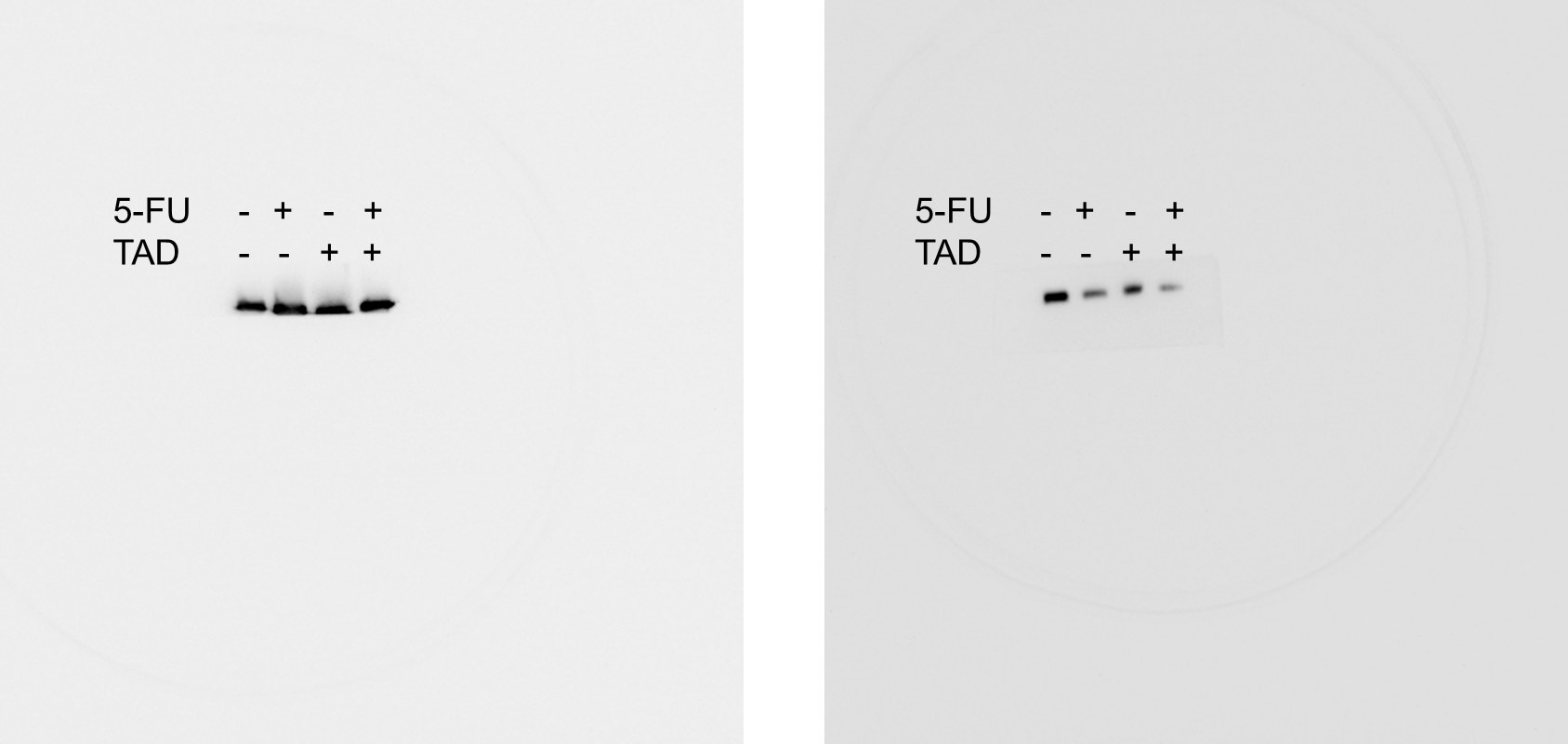


Figure 6F Original blotting of LDHA expression in tumor tissues.

Left: anti-β-actin; Right: anti-LDHA


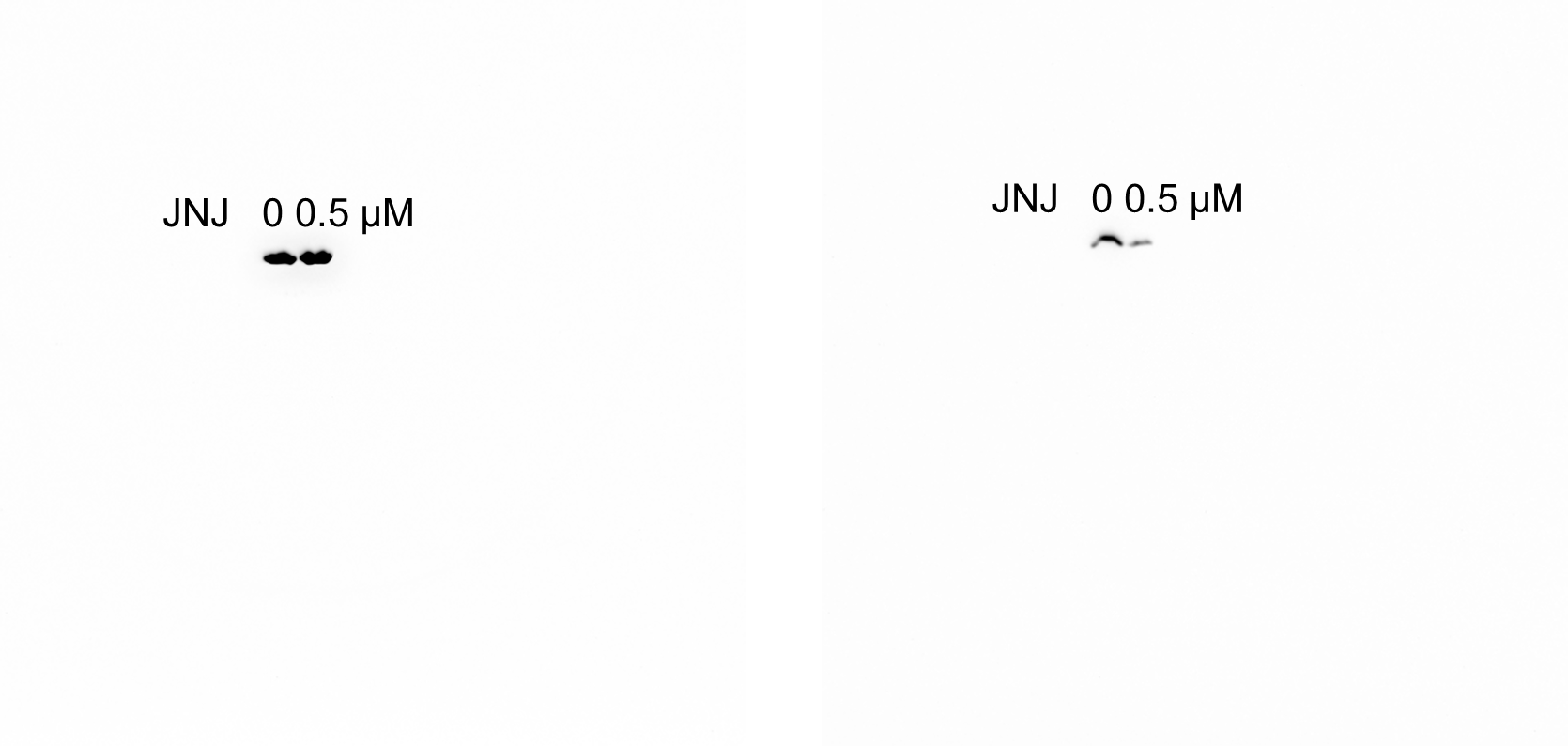


Figure S2C Original blotting of H4R3me2s in HCT116 cells treated with or without JNJ-64619178 (JNJ; 0.5 μM)

Left: anti-β-actin; Right: anti-H4R3me2s


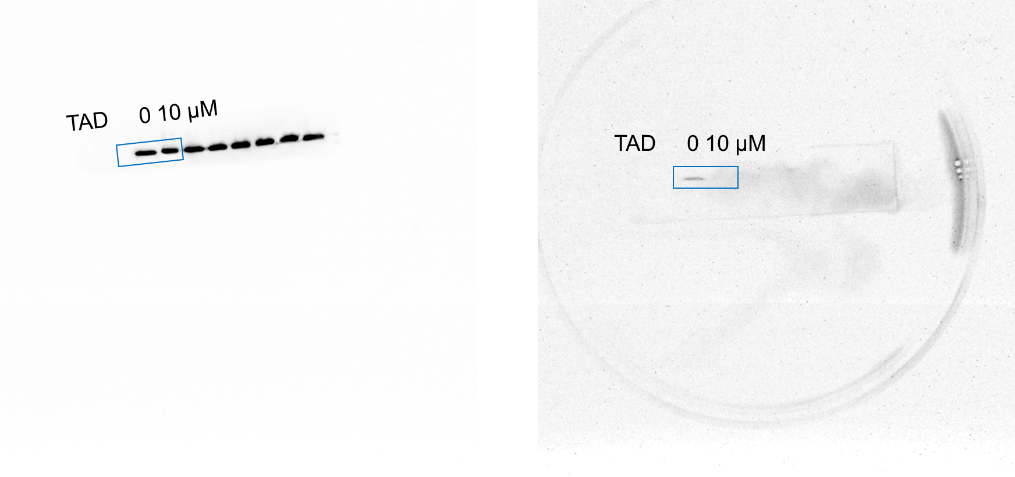


Figure S2D Original blotting of H4R3me2s in HCT116 cells treated with or without tadalafil (TAD; 10 µM)

Left: anti-β-actin; Right: anti-H4R3me2s


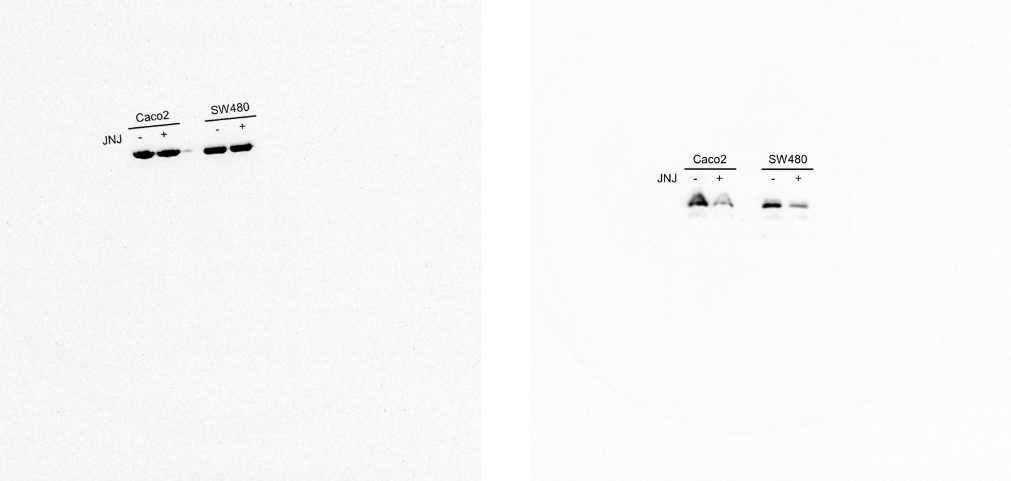


Figure S4G Original blotting of H4R3me2s in SW480 and Caco2 cells treated with or without JNJ-64619178 (JNJ; 0.5 μM)

Left: anti-β-actin; Right: anti-H4R3me2s


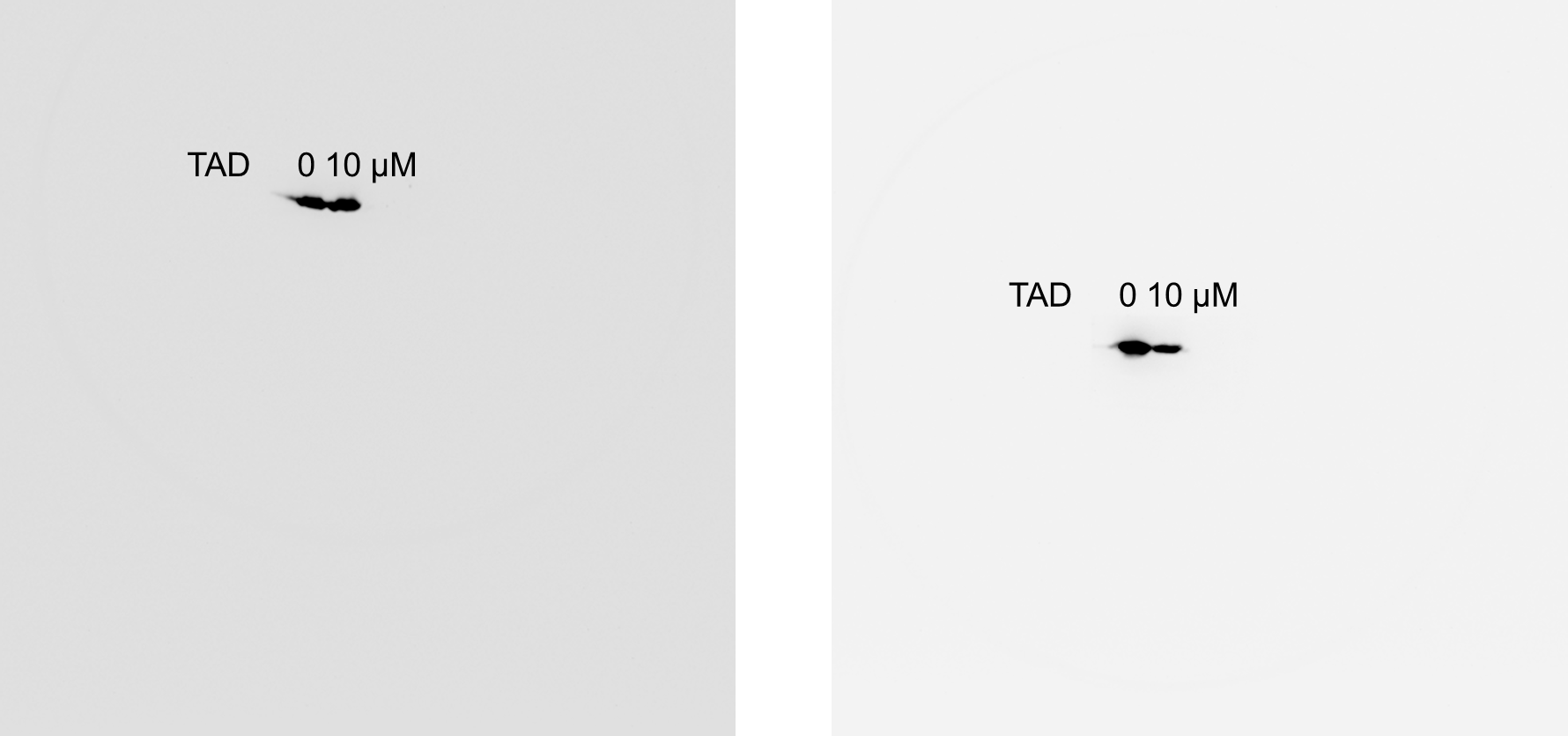


Figure S4H Original blotting of LDHA in HCT116 cells treated with or without tadalafil (TAD; 10 µM)

Left: anti-β-actin; Right: anti-LDHA


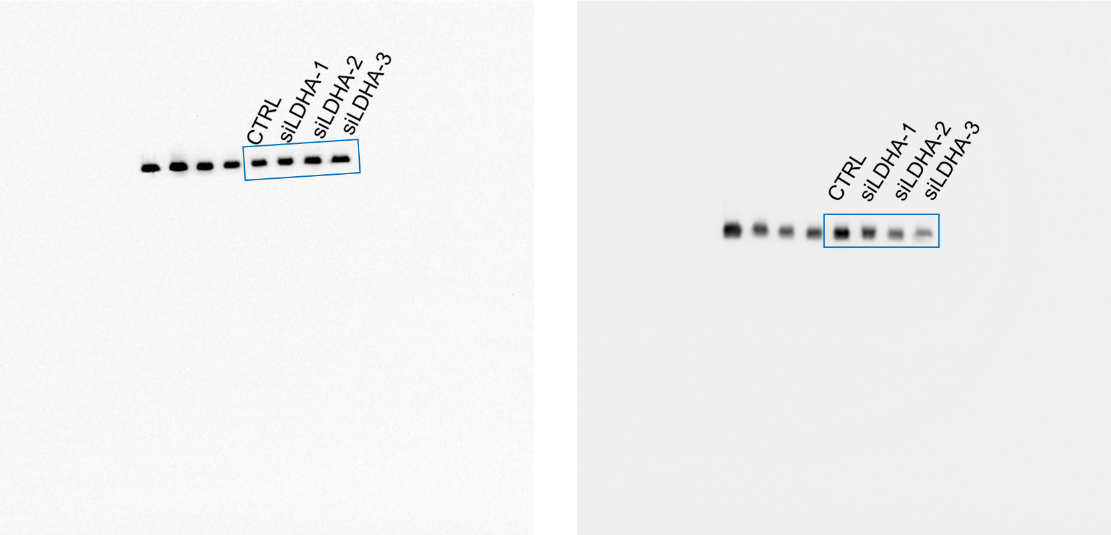


Figure S4I Original blotting of LDHA levels in HCT116 cells transfected with siNC (CTRL), siLDHA-1，siLDHA-2 and siLDHA-3.

Left: anti-β-actin; Right: anti-LDHA
